# Neurocognitive mechanisms underlying deceptive hazard evaluation: An event-related potentials investigation

**DOI:** 10.1371/journal.pone.0182892

**Published:** 2017-08-09

**Authors:** Huijian Fu, Wenwei Qiu, Haiying Ma, Qingguo Ma

**Affiliations:** 1 School of Management, Guangdong University of Technology, Guangzhou, Guangdong, People's Republic of China; 2 Laboratory of Managerial Psychology and Behavior, Guangdong University of Technology, Guangzhou, Guangdong, People's Republic of China; 3 School of Management, Zhejiang University, Hangzhou, Zhejiang, People's Republic of China; 4 Institute of Neural Management Sciences, Zhejiang University of Technology, Hangzhou, Zhejiang, People's Republic of China; 5 Neuromanagement Laboratory, Zhejiang University, Hangzhou, Zhejiang, People's Republic of China; Universita degli Studi di Roma La Sapienza, ITALY

## Abstract

Deceptive behavior is common in human social interactions. Researchers have been trying to uncover the cognitive process and neural basis underlying deception due to its theoretical and practical significance. We used Event-related potentials (ERPs) to investigate the neural correlates of deception when the participants completed a hazard judgment task. Pictures conveying or not conveying hazard information were presented to the participants who were then requested to discriminate the hazard content (safe or hazardous) and make a response corresponding to the cues (truthful or deceptive). Behavioral and electrophysiological data were recorded during the entire experiment. Results showed that deceptive responses, compared to truthful responses, were associated with longer reaction time (RT), lower accuracy, increased N2 and reduced late positive potential (LPP), suggesting a cognitively more demanding process to respond deceptively. The decrement in LPP correlated negatively with the increment in RT for deceptive relative to truthful responses, regardless of hazard content. In addition, hazardous information evoked larger N1 and P300 than safe information, reflecting an early processing bias and a later evaluative categorization process based on motivational significance, respectively. Finally, the interaction between honesty (truthful/deceptive) and safety (safe/hazardous) on accuracy and LPP indicated that deceptive responses towards safe information required more effort than deceptive responses towards hazardous information. Overall, these results demonstrate the neurocognitive substrates underlying deception about hazard information.

## Introduction

Deception is an act by which an individual deliberately withholds the truth and attempts to convince the others to accept the incorrect information as true, typically for attaining some benefit or avoiding a loss[1]. We were taught to always tell the truth since childhood, and even learned a lot from fairy tales that portrayed severe consequences of lying. A well-known victim of such consequences is a shepherd boy from Aesop's Fables who tricked twice the villagers into believing that a danger was arising: wolves were coming at his sheep on the hill. But later on no one trusted him again even if the wolves truly appeared, resulting in a tragic ending with all his sheep killed by the wolves. As a matter of fact, in many cases deceptive acts could induce confusion about the situation and distrust on the liar. Though deception is frequently thought to be morally unacceptable, it is not rare in human social interactions, sometimes for malicious reasons and sometimes for benign ones[2]. Therefore, researchers have gained increasing interest in uncovering the cognitive process and the underlying neural mechanism for deception and truth-telling in the recent decade [3–10].

As Pfister et al. [8] noted, two distinct perspectives with quite different experimental approaches have been adopted by existing researches. The first is concerned with lie detection, a prominent approach of which is Concealed Information Test (CIT, also known as Guilty Knowledge Test, GKT)[11,12]. Typical CITs comprise three types of stimuli: deception-relevant probe stimuli that are known by the subjects who are requested to make incorrect responses in order to conceal their knowledge to these stimuli, target stimuli that are known by the subjects and request honest responses, and irrelevant stimuli that are unknown to the subjects prior to the experiment and require honest responses. Knowledge about the probe stimuli could be inferred if the physiological responses are stronger for probe stimuli than irrelevant stimuli because the concealed information is thought to be meaningful for the subject [11,13,14]. However, results acquired from CIT-based researches do not directly enhance our theoretical understanding of deceptive behavior due to the excessive dependence of the paradigm on recognition of meaningful items [8]. Consequently, many researchers have adopted a second perspective that is concerned with active lying, which requests the subjects to make a truthful or deceptive response towards a presented stimuli [4,6,15,16]. Furedy et al. [17] proposed the Differentiation of Deception Paradigm (DDP), which required the same set of stimuli be answered twice, once truthfully and once deceptively, and thus eliminated the possible confounds brought by stimulus significance and probability. Take Johnson and his colleagues’ work as example, subjects were presented with old and new words with equal probability, and instructed to respond truthfully or deceptively about whether they know the words [3,4,18]. Such a DDP-based old/new paradigm that exploits episodic memory effect has been widely used [19,20].

More recently, researchers have paid close attention to the deceptive process involving evaluation that is likely to occur in daily lives instead of relying on memorially based response conflicts in the old/new paradigm. Dong et al. [15], for instance, presented attractive and unattractive facial photos to the subjects following a cue word, “truthful” or “deceptive”, and instructed them to make a judgment on the attractiveness of the facial stimuli according to the cue. It revealed that deceptive response to attractive faces required a longer RT and triggered higher LPC amplitude than deceptive response to unattractive faces. Moreover, truthful and deceptive behavior concerning the evaluation of attitude[21], emotion [16,22], self-relevance [23] and interpersonal familiarity [9] were investigated, and the moderating roles of the evaluations on truthful and deceptive responses were also observed.

However, so far little attention has been paid to the effect on neurocognitive activity when deception involves hazard information, suggesting a gap in the research. Indeed, deceptions that relate to hazard information are not uncommon, which could sometimes cause drastic consequences. For instance, some promoters might claim their new products or technologies with potential hazard to be absolutely safe, and a parent might prevent his child from attending a safe activity by claiming it to be dangerous. Furthermore, there is well established literature showing that hazardous/risky information is processed differently from safe information in the brain [24–31]. Hazardous/risky information, in contrast to safe information, has been found to elicit stronger activations in medial prefrontal cortex [24,25,31], anterior insula[29,31], ventral anterior cingulate cortex and posterior cingulate cortex[24–26,29], etc. Meanwhile, ERPs studies suggested that hazardous/risky information evoke larger P200 and LPP amplitudes than safe information [26–28]. Therefore, the primary aim of the current study was to uncover the neural correlates of deception when it is associated with hazard evaluation via electrophysiological techniques.

The cue for response type, and the hazard content of the target pictures were manipulated on a trial-by-trial basis in a cue-target paradigm using factorial design. A cue indicating the response condition of each trial (truthful or deceptive) was followed by the presentation of the target, a picture conveying or not conveying hazard information. Participants were asked to discriminate the hazard content of the target picture and make a response according to the cue. At the behavioral level, RT and accuracy merit close attention. According to the prior literature, lying, compared with truth-telling, is consistently associated with longer RT, implicating that lying is cognitively more demanding[7–9,16,23]. Meanwhile, there are different findings concerning response accuracy: some researchers have reported lower accuracy for lying versus truth-telling [18,23], while others haven’t observed such a difference [9,15,16]. ERPs components of interest were the N1, N2, P300 and late positive potential/complex (LPP/LPC). The frontal N1 component, as the first negativity arising around 100ms after stimulus onset, is susceptible to attention orientation at the early stage. Smith et al. [32] observed an enlarged N1 response for unpleasant stimuli than pleasant stimuli over the anterior sites when asked the participants to rate the valence and arousal of emotional pictures. In addition, larger N1 amplitude was evoked by threatening stimuli relative to neutral and positive stimuli, which was thought to index a stronger emotional response and the processing bias for threatening information [33]. The N2, a negative-going component occurs around 200–350 ms over anterior sites, has been hypothesized to play a critical role in cognitive control, and more specifically, in conflict detection[34,35]. Wu et al. [36] asked participants to make deceptive and honest response that were either self-determined or forced, which for the first time observed that lying elicited greater N2 amplitude than truth-telling. N2 amplitude may be increased because lying triggers response conflict between an initial impulse to tell the truth and the opposing objective to lie [7,23]. The P300 is a positive component peaking at approximately 300–400 ms, distributed from frontal to parietal scalp sites and typically maximal over central-parietal regions. Motivationally significant stimuli have been found to elicit greater P300 component [37]. For instance, larger P300 amplitude was evoked by emotional stimuli compared to neutral stimuli, reflecting increased allocation of attentional resources based on emotional significance[38,39]. Moreover, P300 is associated with evaluative categorizations, which could differentiate emotional or threatening stimuli from neutral stimuli during active evaluation [40,41]. The LPP/LPC, a positivity belonging to the P300 family, generally arises at about 400 ms and lasts for several hundred milliseconds. As Johnson et al. [4] and Dong et al. [15] noted, an attenuated LPC was induced by deceptive relative to truthful responses, suggesting the inhibition of truthful responses and the increase of cognitive load for preparing deceptive responses, which drew the processing resources away from the primary task of responding truthfully.

In sum, the current study attempted to extend previous deception results by investigating the behavioral and electrophysiological correlates of deception about hazard information. In accordance with previous studies, behaviorally, we hypothesized a longer RT and lower accuracy for deceptive than for truthful responses. With regard to the neural correlates, we expected the following: (1) an increased N2 and an attenuated LPP for deceptive versus truthful responses, (2) a more negative N1 and an increased P300 for hazardous items compared with safe items, and (3) lying about hazardous and safe items would be distinct from each other, which was reflected by the LPP.

## Method

### Participants

Twenty-two undergraduates or graduate students (4 females), ranging in age from 20 to 30 years (Mean±SD = 22.94±1.95), were recruited from Zhejiang University. All subjects were right-handed, had normal or corrected-to-normal vision, and reported no history of neurological disorders or mental diseases. The experiment conformed to the Declaration of Helsinki and was approved by the Neuromanagement Laboratory Ethics Committee at Zhejiang University. All subjects gave written informed consent prior to the experiment and were paid for their participation. Data from three subjects were discarded, two for excessive artifacts during EEG recording and one for misunderstanding the rules of the task, resulting in nineteen valid subjects for the final data analysis.

### Materials

The stimuli were 20 safety signs or public information graphical symbols selected according to the recommendation list of the Chinese National Standard [42,43]as they are clearly discernible in terms of whether they convey hazard information. All pictures were processed by Photoshop software (Adobe Photoshop 13.0; Adobe Systems Incorporated, San Jose, California, USA) to ensure consistency in background, brightness, contrast and color saturation, and adjusted to a uniform size (561 by 472 pixels). Based upon previous studies on safety warnings [28,44,45], the pictures were classified by the strength of perceived hazard into a hazardous group with high hazard level and a safe group with low hazard level (10 pictures in each group). To confirm the classification, twenty-three subjects who did not participate in the electrophysiological experiment rated the strength of perceived hazard of the pictures using a seven-point Likert scale (1 = very low, 7 = very high). Paired t-test showed a significant difference in the mean hazard levels between the two groups (hazardous group = 5.80, safe group = 3.16; t(22) = 9.466, p = 0.000). The alpha reliability coefficients were 0.775 and 0.944 for hazardous and safe groups, respectively.

### Procedure

**Fig 1:**
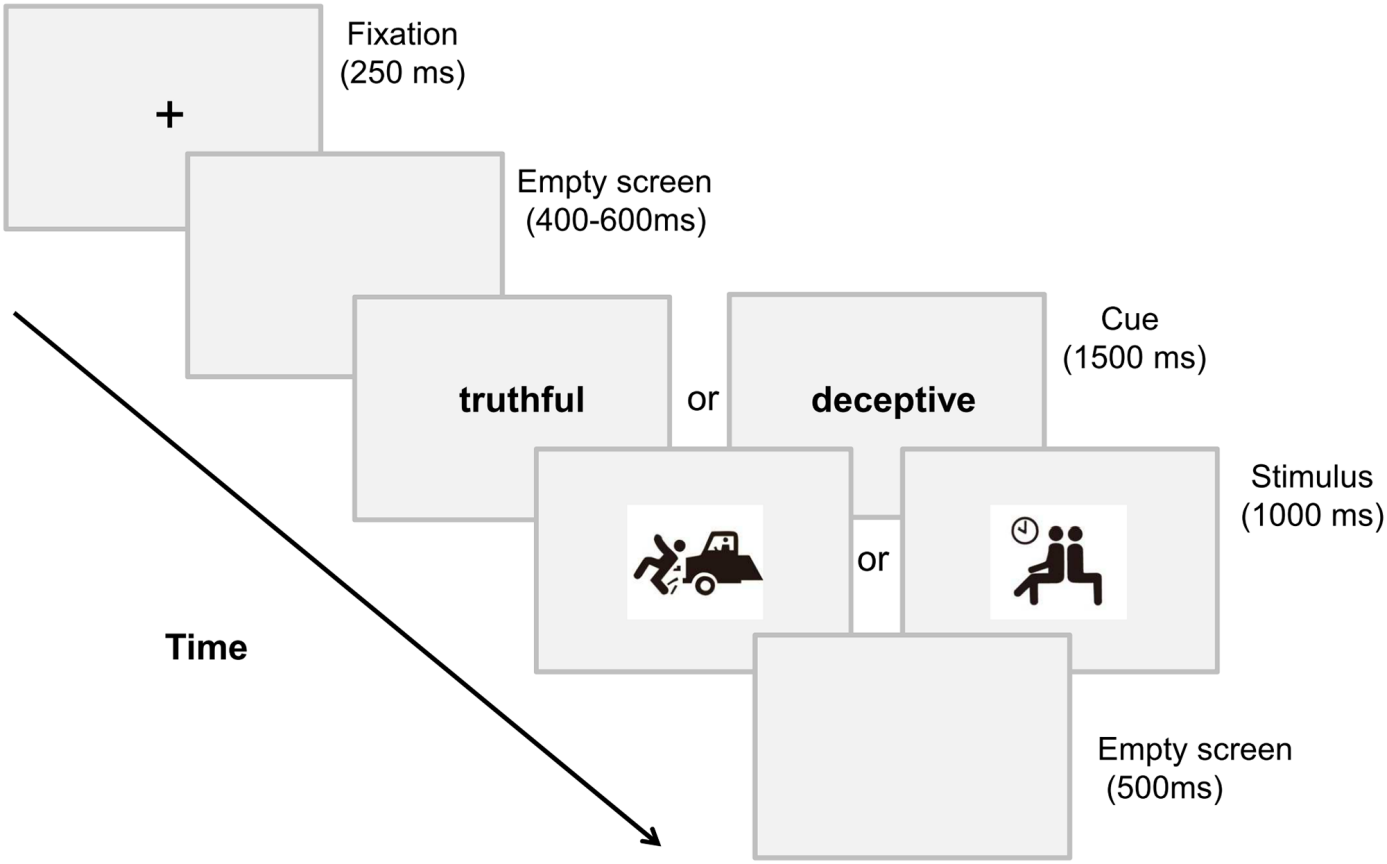
A single trial of the experimental procedure. Subjects first saw a cue word “truthful” or “deceptive” before the presentation of a picture stimulus, and were instructed to make responses (“safe” or “hazardous”) on the picture stimulus according to the cue.

Subjects were comfortably seated in a dim, sound-attenuated and electrically shielded room to complete two blocks of trials. A keypad was provided to the subjects to make responses. The experimental procedure was introduced on paper handouts and the subjects were challenged to lie as skillfully as they could to deceive our experimenter. Before the formal experiment, each subject had 20 practice trials to become familiar with the experimental procedure. Stimuli were delivered by E-Prime 2.0 (Psychology Software Tools Inc., Pittsburgh, PA, USA) and presented on the computer screen against a grey background at a distance of 90 cm from the subject. As illustrated in Fig 1, in each trial, a central fixation cross was presented for 250 ms, followed by an empty screen for a random duration of 400–600 ms. Then, a cue word, “truthful” or”deceptive” (in Chinese), appeared at the center of the screen for 1500ms, after which a picture embodying or not embodying hazard information was presented with a visual angle of 6.621°×5.093°. The subjects were asked to make a corresponding response to the picture stimulus according to the cue. In truthful condition, subjects were requested to make a truthful “safe” or “hazardous” judgment on the picture; while in deceptive condition, they were instructed to make a deceptive “safe” or “hazardous” judgment by giving an opposite response. Therefore, four experimental conditions (i.e., truthful/safe, truthful/hazardous, deceptive/safe and deceptive/hazardous) were included in this task. The subject had to respond as soon as possible, and the response deadline was set to 1500 ms. Response keys were counterbalanced among the subjects such that half of them were required to press “1” for hazardous and “3” for safe while the other half received opposite instructions. After the picture stimulus vanished, an inter-trial interval lasted for 500 ms. In each condition, there were 10 different pictures, each of which was shown for four times; thus, there were 160 trials altogether. The order of the trials was random within each block.

### EEG data acquisition

Electroencephalogram (EEG) was recorded with an electrode cap with 64 Ag/AgCl electrodes mounted according to the extended international 10–20 system, with online band-pass-filtered from 0.05 to 100 Hz, and sampled at 1000 Hz using Neuroscan Synamp2 Amplifer (Scan 4.5, Neurosoft Labs, Inc. Sterling, USA). Electrooculogram (EOG) was recorded from electrodes placed 1.0cm lateral to the external canthi of both eyes (horizontal EOG), and above and below the left eye (vertical EOG). The electrode on the cephalic region was applied as the ground. The left mastoid served as on-line reference, and EEGs were off-line re-referenced to the algebraically computed average of the left and right mastoids. Impedances were maintained below 5kΩ throughout the experiment.

### Data reduction and analysis

For the behavioral data, two-way repeated measures ANOVAs were conducted to compare RT and accuracy across four conditions, 2(honesty: truthful, deceptive) × 2(safety: safe, hazardous). Only correct responses were included for RT analysis.

For the ERP data, during the offline EEG analysis, vertical and horizontal EOG artifacts were corrected using the method proposed by Semlitsch et al.[46], followed by digital filtering through a zero phase shift (low pass at 30 Hz, 24 dB/octave). Stimulus-locked data were segmented into epochs comprised of 200 ms before stimulus onset and 1000 ms after the onset. The entire epoch was then baseline-corrected by the 200ms interval prior to the stimulus onset. Trials containing amplifier clipping, bursts of electromyography activity, or peak-to-peak deflection exceeding±100 μV were excluded from averaging. Among all participants, more than 30 valid trials for each condition remained and entered the data processing, which are suggested to be sufficient to achieve stable and reliable measurements of Event-related potentials[47]. The recorded EEGs over each recording site for each participant were averaged separately within each of the experimental conditions.

Based on visual observation of the grand average waveforms as well as previous ERP studies on deception [7,8,15,23] and hazardous/risky information processing [26,27], four ERP components—N1, N2, P300 and LPP—were analyzed. Six electrodes (F3, FZ, F4, FC3, FCZ and FC4) in the frontal-central areas were selected for N1 and N2 analysis, nine electrodes (C3, CZ, C4, CP3, CPZ, CP4, P3, PZ and P4) in the central-parietal areas were selected for P300 analysis, and fifteen electrodes (F3, FZ, F4, FC3, FCZ, FC4, C3, CZ, C4, CP3, CPZ, CP4, P3, PZ and P4) distributed from the frontal to parietal areas were selected for LPP analysis. ERP amplitudes in the time windows of 80-130ms, 220-250ms, 280-400ms and 430-700ms post–onset of the target stimuli were averaged for N1, N2, P300 and LPP analysis respectively. The amplitude values of the N1 and N2 were separately submitted to 2 (honesty: truthful, deceptive) × 2 (safety: safe, hazardous) × 6 (electrodes: F3, FZ, F4, FC3, FCZ, FC4) repeated measure ANOVAs. Similar analyses were performed for P300 with nine electrodes and LPP with fifteen electrodes included. The Greenhouse-Geisser correction was applied in all statistical analysis when necessary (uncorrected df and corrected p-value were reported). A simple effect analysis was conducted when the interaction effect was significant.

In addition, correlation analyses were performed at the group level in order to explore if there were functional connections between one’s brain activity and behavioral performance, and more specifically, between the mean amplitude of LPP and RT/accuracy. We defined two contrasts corresponding to the simple main effect of honesty (deceptive/safe—truthful/safe, deceptive/hazardous—truthful/hazardous) and an interaction contrast [(deceptive/safe—truthful/safe)–(deceptive/hazardous—truthful/hazardous)]. The values for LPP, RT and accuracy were computed according to these contrasts before entering the two-tailed Pearson correlation analyses.

## Results

### Behavioral results

**Fig 2:**
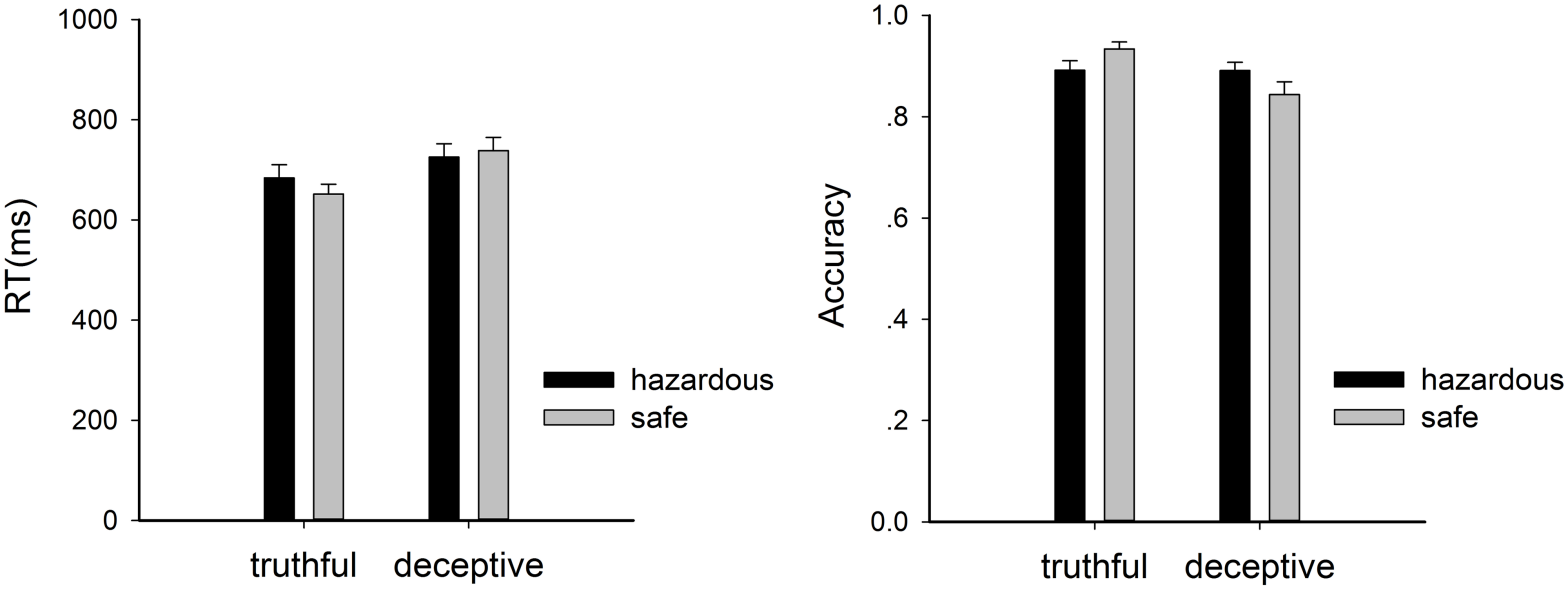
RT and accuracy for each condition. The error bars denote standard error of the mean.

The ANOVA analysis for RT (Fig 2) revealed a main effect of honesty (F_1,18_ = 54.325, p = 0.000, η^2^_partial_ = 0.751), but not safety (F_1,18_ = 0.732, p = 0.403, η^2^_partial_ = 0.039). Generally, the RT for a deceptive response (M = 732.12ms, S.D. = 113.21) was longer than that for a truthful response (M = 668.02ms, S.D. = 93.66). The interaction effect between honesty and safety was significant (F_1,18_ = 7.469, p = 0.014, η^2^_partial_ = 0.293), and the simple effect analysis demonstrated that it took much longer for a deceptive response relative to truthful response in both safe and hazardous conditions (ps≤0.001), but the difference between deceptive-to-safe and truthful-to-safe items (mean difference = 86.74 ms) was significantly larger than that between deceptive-to-hazardous and truthful-to-hazardous items (mean difference = 41.45 ms) (t = 2.733, p = 0.014).

A similar pattern was observed for response accuracy, which also showed a main effect of honesty (F_1,18_ = 13.746, p = 0.002, η^2^_partial_ = 0.433) but not safety (F_1,18_ = 0.044, p = 0.836, η^2^_partial_ = 0.002). The accuracy for deceptive responses (M = 0.867, S.D. = 0.082) was lower than that for truthful responses (M = 0.913, S.D. = 0.062). Moreover, a significant interaction between honesty and safety was observed (F_1,18_ = 10.220, p = 0.005, η^2^_partial_ = 0.362), and the simple effect analysis demonstrated that the accuracy was lower for truthful-to-hazardous than truthful-to-safe items (F_1,18_ = 5.914, p = 0.026, η^2^_partial_ = 0.247), but higher for deceptive-to-hazardous than deceptive-to-safe items (F_1,18_ = 5.554, p = 0.030, η^2^_partial_ = 0.236).

### ERP results

**Fig 3:**
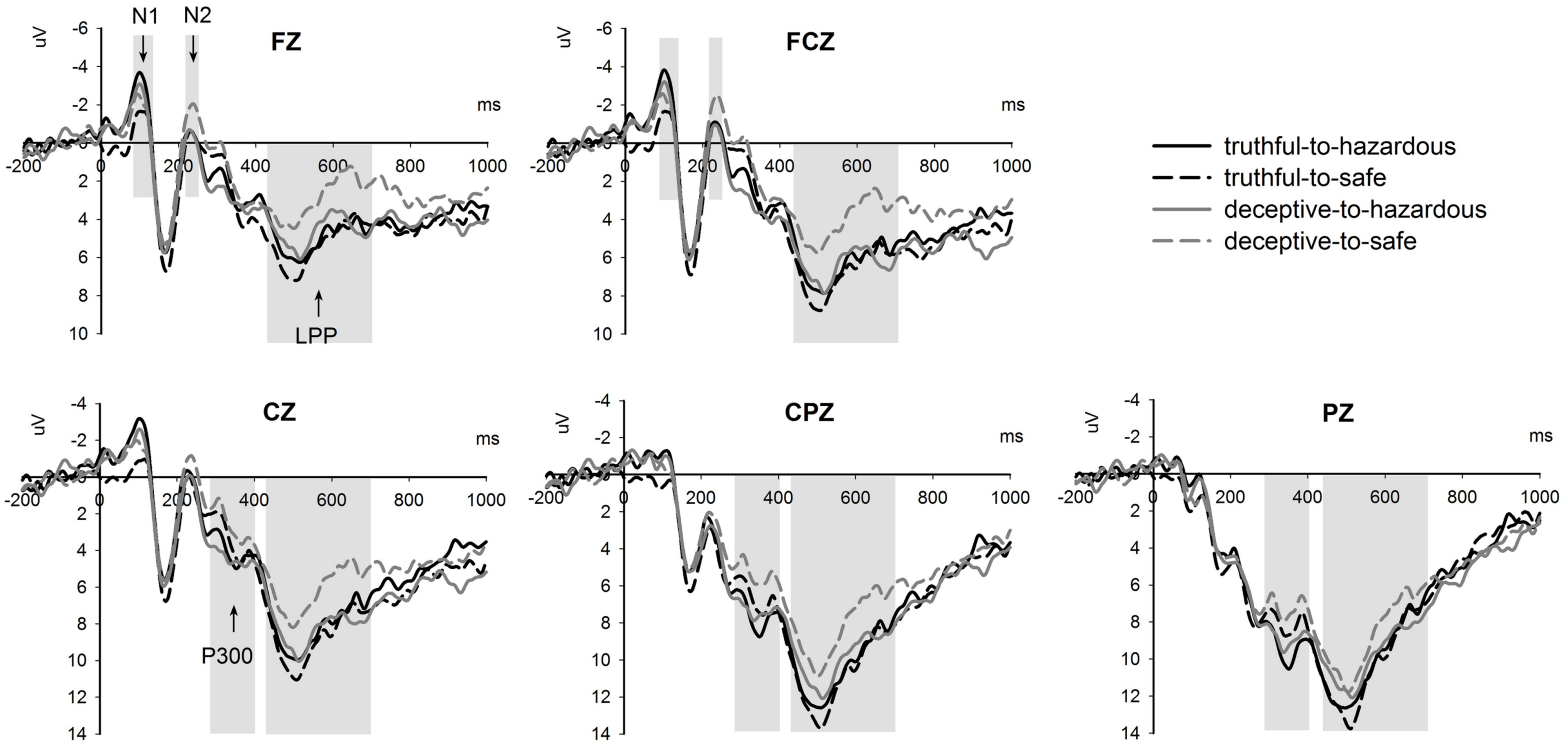
The grand average waveforms at channel FZ, FCZ, CZ, CPZ and PZ showing the potentials produced in response to the presentation of the target stimulus. Positive voltage is plotted downwards.

**Fig 4:**
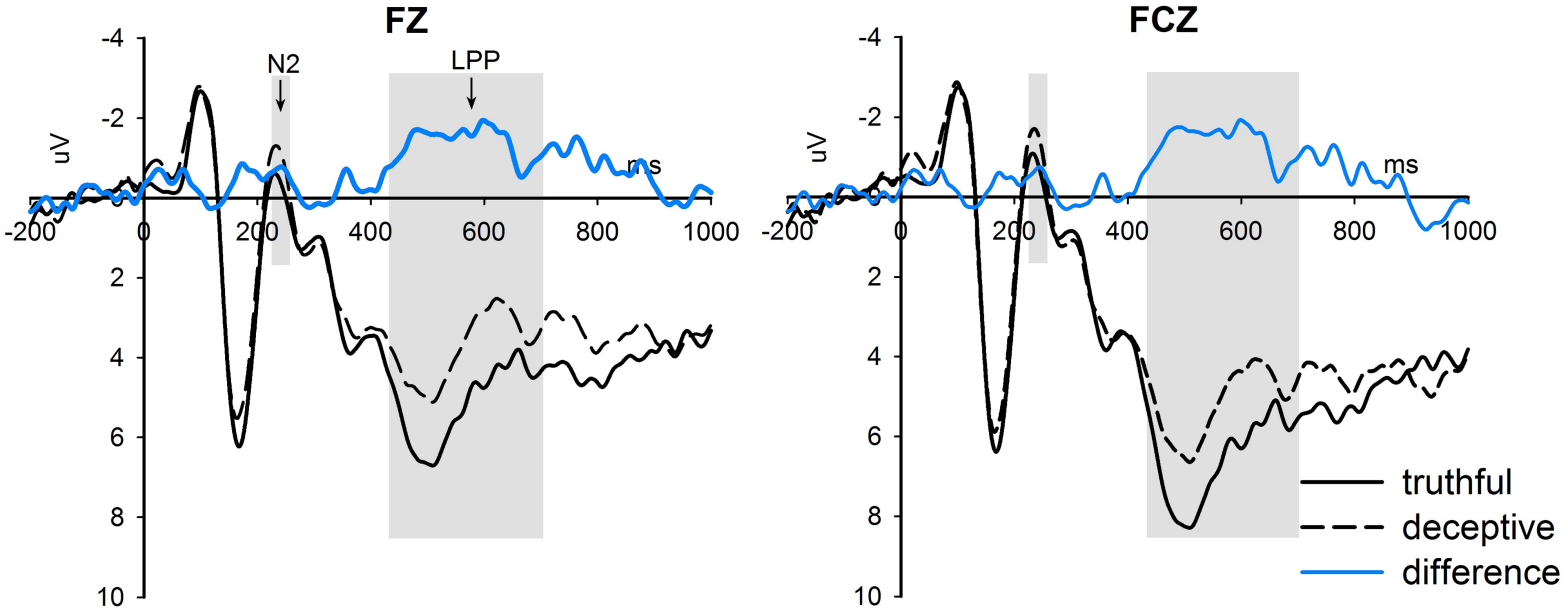
The difference waveforms between deceptive and truthful conditions at frontal electrodes FZ and FCZ.

The ERP grand average waveforms at the middle electrodes FZ, FCZ, CZ, CPZ and PZ are presented in Fig 3. For a clear overview, the results of the three-way repeated measure ANOVAs on mean amplitudes of the N1, N2, P300 and LPP components are summarized in Table 1. In general, the deceptive condition evoked augmented N2 and reduced LPP components relative to the truthful condition (for N2: M_deceptive_ = -0.232 μV, S.D._deceptive_ = 4.916; M_truthful_ = 0.429 μV, S.D._truthful_ = 5.064; for LPP: M_deceptive_ = 6.524μV, S.D._deceptive_ = 3.092; M_truthful_ = 7.719 μV, S.D._truthful_ = 3.167). The difference waveforms between deceptive and truthful conditions at frontal electrodes FZ and FCZ are displayed in Fig 4. Meanwhile, the hazardous condition elicited increased N1 and P300 amplitudes relative to the safe condition (for N1: M_hazardous_ = -2.264 μV, S.D._hazardous_ = 1.912; M_safe_ = -1.400 μV, S.D._safe_ = 1.842; for P300: M_hazardous_ = 7.259μV, S.D._hazardous_ = 3.430; M_safe_ = 6.116 μV, S.D._safe_ = 3.012).

**Table 1:** Results of the ANOVAs on mean amplitudes for N1, N2, P300 and LPP components.

		N1 (80-130ms)	N2 (220-250ms)	P300 (280-400ms)	LPP (430-700ms)
Honesty	F	0.042	4.738*	2.592	10.750**
Safety	F	9.739**	1.338	6.414*	2.831
Electrodes	F	5.429*	17.213***	11.836***	14.266***
Honesty × Safety	F	4.571*	3.225	1.945	5.471*

For Honesty, Safety and Honesty × Safety, for all components, df = (1, 18). For Electrodes, for N1 and N2, df = (5, 90); for P300, df = (8, 144); for LPP, df = (14, 252).

*p <0.05,

**p<0.01,

***p<0.001.

Since the analyses on the N1 and LPP revealed significant interactions between honesty and safety, simple effect analyses were performed respectively for these two components. For the N1 component, truthful-to-hazardous items (M = -2.530 μV, S.D. = 2.090) elicited a more negative deflection than truthful-to-safe items (M = -1.092 μV, S.D. = 1.939) (F_1,18_ = 15.039, p = 0.001, η^2^_partial_ = 0.455), yet deceptive-to-hazardous and deceptive-to-safe items didn’t show a difference (F_1,18_ = 0.522, p = 0.479, η^2^_partial_ = 0.028). For the LPP component, deceptive-to-hazardous items (M = 7.307 μV, S.D. = 3.158) induced a more positive deflection compared with deceptive-to-safe (M = 5.742μV, S.D. = 3.323) (F_1,18_ = 12.268, p = 0.003, η^2^_partial_ = 0.404), but no difference was found between truthful-to-hazardous and truthful-to-safe items (F_1,18_ = 0.358, p = 0.557, η^2^_partial_ = 0.019).

### Correlation analyses results

**Fig 5:**
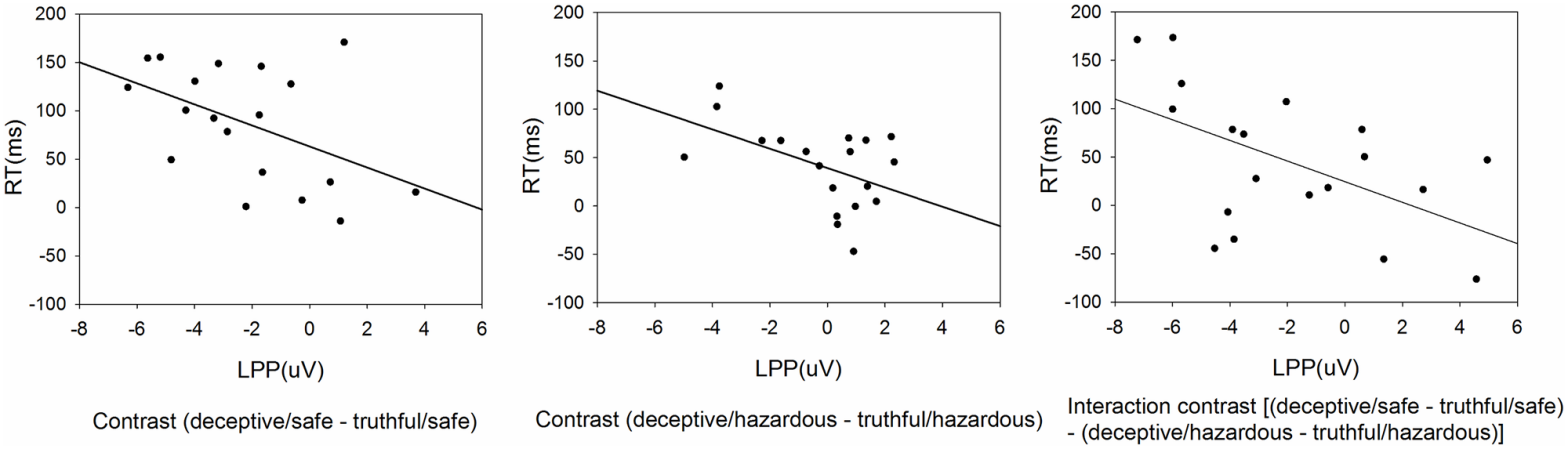
Correlations between LPP amplitude and RT. The values for LPP and RT were computed according to the contrasts before entering the two-tailed Pearson correlation analyses.

Three contrasts of interest were performed separately, all of which showed significant negative correlations between LPP amplitude and RT (Fig 5): for the contrast (deceptive/safe—truthful/safe), r = -0.479, p = 0.038; for the contrast (deceptive/hazardous—truthful/hazardous), r = -0.495, p = 0.031; for the interaction contrast [(deceptive/safe—truthful/safe)—(deceptive/hazardous—truthful/hazardous)], r = -0.535, p = 0.018. However, no significant correlations were found between LPP amplitude and accuracy.

## Discussion

In the present study, we explored the behavioral and electrophysiological correlates of deception about hazard information using a cue-target experimental paradigm. Results showed that deceptive responses, in contrast to truthful responses, were associated with longer RT, lower accuracy, increased N2 and reduced LPP. Further analysis revealed negative correlations between the increments in LPP amplitude and the decrements in RT for deceptive versus truthful responses, regardless of hazard content. Meanwhile, hazardous items evoked larger N1 and P300 than safe items. Additionally, interaction effects between honesty and safety were observed on RT, accuracy, N1 and LPP.

Behaviorally, the prolonged RT and reduced accuracy suggest that deception entails more conflict and control, coinciding with previous studies using a similar paradigm [7–9,16,23]. Therefore, deception is a cognitively more demanding process than truth-telling. In order to tell a lie, one has to recognize what is true (the first step) and ensure that the actual response is different from the truthful one (the second step). The second step might result in extra processing time and reduced accuracy [8]. The interaction of honesty and safety on RT revealed larger RT difference between deceptive-to-safe and truthful-to-safe items versus that between deceptive-to-hazardous and truthful-to-hazardous items, suggesting it much more cognitively demanding to lie than tell the truth when the information is safe. Moreover, hazardous information is retrieved with more ease and processed more deeply than safe information in the deceptive condition, as evidenced by the higher accuracy for deceptive-to-hazardous instead of deceptive-to-safe items.

The main effect of honesty on neural correlates showed more pronounced N2 and LPP components for deceptive responses. N2 has been found to play a critical role in the detection of conflict [34,35]. Wu et al. [36], for the first time, observed a larger N2 for lying than truth-telling when they asked participants to make deceptive or honest response with a revised old/new paradigm. The response conflict between an initial impulse to tell the truth and the opposed objective to lie may give rise to the N2 deflection [23,36]. Consistent with most prior work, given its spatial-temporal features and sensitivity to deception, the N2 here might represent the conflict detection and response inhibition process, implying that more executive control process is required for deceptive responses[7,23,34,36]. However, an exceptional finding was reported by Pfister et al. [8], which showed enlarged N2 for truthful responses rather than deceptive responses when the participants were asked to indicate the location of a knife truthfully or deceptively. They assumed that N2 was functionally linked to P300 and sensitive to general processing demands. A likely interpretation for the divergence between Pfister et al. [8] and the present study might be that, in their study, the participants could freely choose whether to lie in each trial before the appearance of the target, which didn’t seem to pose a noticeable degree of response conflict to the participants when executing a deceptive response.

The LPP, an obvious component of P300 family, was observed at 430–700 ms. Due to its occurrence in tasks involving stimulus evaluation and memory updating, LPP is often associated with elaborate processing related to stimulus significance, such as memory encoding, storage and retrieval [48,49]. The cognitive load hypothesis in lying suggests a dual task character of lying, with different tasks requiring division of attention [7,50]. In a pioneering study on the electrophysiological signature of active lying, a reduced LPC was observed for deceptive responses relative to truthful responses, implicating that the monitoring and inhibition of truthful responses induced higher demand on cognitive processing and drew attentional resources away from the primary task of responding truthfully so as to make a deceptive response[4]. Similarly, a large number of studies have shown attenuated P300 amplitude for lying compared to truth-telling [3,7,23,36]. It’s worth noting that the LPP from the current study reflects the same cognitive processes as the P300 from those studies since they had similar scalp distribution, arose at around 400ms and sustained for several hundred milliseconds, and most importantly, were sensitive to deception. In line with previous studies, the attenuated LPP here indicates that attentional resources are drawn away from the primary task of responding truthfully in order to inhibit the truth and execute a deceptive response. The longer RT and lower accuracy for deceptive versus truthful responses also support this interpretation. More importantly, the decrements in LPP amplitude correlated negatively with the increments in RT for deceptive relative to truthful responses, no matter for safe or hazardous items, suggesting possible functional connections between neurocognitive activity and corresponding behavioral performance. The more the LPP decreases, the more the RT increases. As the amplitude of LPP is well recognized to reflect the available attentional resources, which decrease in proportion to the amount of resources allocated to secondary tasks (e.g., responding deceptively)[3,4,7,15,21], this finding suggest that those participants who exhibited more LPP reduction needed to devote greater effort to completing deceptive responses and correspondingly, showed more reduction in behavioral efficiency (i.e., a higher increase in RT).

The main effect of safety showed N1 and P300 differentiations between safe and hazardous items. A more negative N1 for hazardous versus safe items indicates a stronger emotional response towards hazardous items at a relatively early processing stage. Previous studies have reported enlarged N1 responses for unpleasant stimuli compared to pleasant stimuli over the anterior sites [32], and for threatening stimuli relative to neutral and positive stimuli[33]. Such processing bias ensures that processing resources are more readily oriented towards evolutionary significant information [37]. However, the N1 differentiation between safe and hazardous items was found only for truthful condition. We speculate that the objective to lie inflicts higher cognitive load on the participants, which reduces the resources available for early processing. With regard to P300, it has been hypothesized to reflect task-related categorization and higher-order processes that demand attentional resources for evaluating and categorizing stimuli [51]. Correll et al. [40]engaged participants in a video game in which they had to discriminate whether the man in a photo was armed, and found augmented P300 for armed compared to unarmed targets. Studies using emotional, risky and aesthetics stimulus with active judgment tasks have shown similar P300 modulations[26,41,52]. In the present study, hazardous items are safety signs conveying potential hazard information, which is crucial for human survival and preservation; therefore, hazardous items are evolutionarily more salient than safe items. Hence the increase in P300 amplitude for hazardous versus safe items might indicate that, hazardous items, with stronger motivational significance, mobilizes more attentional resources during hazard evaluation and categorization. Such an evaluation and categorization process is crucial in this task because the participants have to find out what is true before making an actual response corresponding to the cue.

Interestingly, interaction effects were found between honesty and safety in LPP component. Deceptive-to-safe items triggered reduced LPP amplitude compared to deceptive-to-hazardous items. According to the prior discussion on LPP, the decrement in LPC might signal the inhibition of truthful responses in order to perform a deceptive response [4,15]. Thereby we suppose that it requires more effort to inhibit the truthful responses when lying about safe compared to hazardous items. A possible explanation might be that hazardous items, given its stronger motivational relevance, recruit more sustained attentional resources and is more elaborately processed relative to safe items, which enhance the efficiency of the cognitive process and make it less difficult to respond deceptively. Previous studies have provided numerous assertions that evolutionarily salient information is more likely to evoke the motivation system and obtain sustained high-level attentional processing, which could be indicated by increased LPP amplitude[37,38,48,53]. Schupp et al. [53], for instance, asked the participants to view emotional pictures and observed larger LPP for stimuli depicting scenes of threat and mutilation than neural stimuli, which was indicative of heightened attention to stimuli that activate defensive motivational system. Similarly, emotionally arousing stimuli has consistently been found to induce augmented LPP component[27,38,39,48,54]. Therefore, we speculate that the larger LPP amplitude for deceptive-to-hazardous versus deceptive-to-safe items represents the enhanced attentional processing triggered by the stronger motivational significance of hazardous stimuli, which makes it easier to inhibit the truthful responses and make opposite responses. In consonance with the ERPs result, lower accuracy for deceptive-to-safe instead of deceptive-to-hazardous items also confirms it more difficult to respond deceptively towards safe items than hazardous items. Further analysis on the interaction contrast showed negative correlation between LPP amplitude and RT, which indicated that the interaction between honesty and safety on LPP might be functionally responsible for the interaction on RT.

Although the DDP has been widely used by deception studies in laboratory settings[3,4,9,15,16,18], the limitation to our current protocol is notable. Since the deceptive responses were directed but not spontaneous, we might have identified only a subset of the neurocognitive processes engaged in deception in real life, i.e., those specifically involving in inhibiting a prepotent truthful response and engendering its opposite. Those neurocognitive processes might be mainly activated by the response reversal, but such a response reversal is inherent in a lying scenario[6]. Apart from response reversal, the decision process leading to spontaneous deception also merits close attention [2,55]. Therefore, though the findings of the current study are informative, it would be helpful to consider spontaneous deception of hazard information in future studies.

## Conclusion

To summarize, by asking participants to perform hazard judgment tasks with a cue-target paradigm, the current findings suggest that deceptive responses are more cognitively demanding than truthful responses, as indexed by longer RT, lower accuracy, enlarged N2, and decreased LPP component. The decrements in LPP amplitude correlated negatively with the increments in RT for deceptive relative to truthful responses regardless of hazard content. In addition, larger N1 and P300 were elicited by the hazardous items instead of safe items, indicating an early processing bias and a later evaluative categorization based on motivational significance, respectively. More importantly, the interaction between deception and hazard content in accuracy and LPP component implicates it more effortful to respond deceptively towards safe information than hazardous information. It extends and complements previous deception results, which further proves that deceptive responses are dependent on the content of lying, and more specifically, hazard information in the current study.

## Supporting information

S1 File RAR file containing raw experimental data. (RAR)
