# Correction: Neurocognitive mechanisms underlying deceptive hazard evaluation: An event-related potentials investigation

**DOI:** 10.1371/journal.pone.0213604

**Published:** 2019-03-05

**Authors:** Huijian Fu, Wenwei Qiu, Haiying Ma, Qingguo Ma

In the Funding section, the grant number from the funder National Project is listed incorrectly. The correct grant number is: AWS14J011.
